# Un‐Matched 1:1 Retrospective Case–Control Study of the Transmission of Typhoid Fever in Neno District, Malawi

**DOI:** 10.1002/puh2.70136

**Published:** 2025-10-11

**Authors:** Priscilla Talabuku, Atusaye Mwalwanda, Elton Chavura, Tawonga Mwase‐Vuma, Balwani Chingatichifwe Mbakaya

**Affiliations:** ^1^ Public Health Department University of Livingstonia Livingstonia Malawi; ^2^ Department of Community Development Luke International Mzuzu Malawi; ^3^ Faculty of Medicine Health and Life Science, Swansea University Swansea Wales UK; ^4^ Center For Social Research University of Malawi Zomba Malawi; ^5^ School of Medicine University College Dublin Dublin Ireland

**Keywords:** case–control, Malawi, Neno District, transmission, typhoid fever

## Abstract

Despite multidisciplinary efforts to control typhoid fever, outbreaks continue to recur in Neno District. This study aimed to identify factors contributing to typhoid transmission and explore community knowledge and empowerment strategies. The study comprised of 200 cases and 200 controls. Mixed‐method approach was used. Quantitative data were analyzed with Statistics Training Analysis Translation Assistance (STATA) version 17 to determine associations. Associations between variables were assessed using odds ratios (ORs) to measure the strength of associations. Logistic regression analysis was employed to calculate adjusted OR. Qualitative data were analyzed through thematic analysis. The study revealed that 14.8% of participants lacked knowledge about how typhoid fever spreads, although 85% were aware of preventive measures. Nearly a third of participants (31.5%; *n* = 126), comprising both cases and controls, identified contaminated food and water as the primary sources of transmission. Young adults aged 21–34 years were particularly at risk (OR: 2.8; 95% confidence interval [CI]: 1.0–7.9, *p* = 0.042) of typhoid fever. Eating at restaurants (OR: 3.43; 95% CI: 1.1–10.6, *p* = 0.032), ready‐to‐eat meals sold along the roads (OR: 3.04; 95% CI: 1.3–7.18, *p* = 0.011), storing water in plastic buckets (OR: 7.98; 95% CI: 3.6–17.6, *p* = 0.001), and collecting water less than 30 m from latrines (OR: 7.01; 95% CI: 3.8–12.8, *p* = 0.001) were statistically significantly associated with typhoid fever. Health education, community involvement, and fostering community action were considerably identified as pivotal community empowerment strategies in preventing and controlling typhoid fever. The findings reveal varying gaps in community knowledge about typhoid fever with likely significant opportunities for health education, community involvement, and proactive action, particularly among young adults (youths) aged 21–34 years. Improved water storage and treatment and improved sanitation and food hygiene practices, as well as community knowledge and empowerment strategies, appear to be critical to reducing transmission of typhoid fever in this study area.

## Graphical abstract

This infographic highlights typhoid fever risks, including eating at restaurant, storing water in plastic buckets, and collecting water near latrines. It shows that 14.8% lacked knowledge on transmission, with young adults (21–34 years) at higher risks. Recommendations stress better improved water storage, treatment, sanitation, food hygiene, and community awareness for prevention.

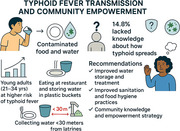

**Abbreviations**
CI: confidence interval
DHO: District Health Office
HMIS: Health Management and Information System
OR: odds ratio
RDT: rapid diagnostic tests
*S*. Typhi: *Salmonella* Typhi
STATA: Statistics Training Analysis Translation Assistance
UNILIAREC: University of Livingstonia Research Ethics Committee
WHO: World Health Organization

## 1 Background Information

Typhoid fever, a challenging illness caused by the bacterium *Salmonella* Typhi (*S*. Typhi), is a persistent and life‐threatening concern that continues to cast a long shadow over regions with limited access to clean water and proper sanitation facilities [1]. This infectious disease exerts a significant impact, particularly in low‐ and middle‐income countries, where the burden is disproportionately high and sub‐Saharan Africa stands as one of the most severely affected regions [1, 2]. The infection is usually transmitted through the ingestion of contaminated food and water sources [3] and usually presents with fever, headache, and abdominal pain; however, multiple severe complications can occur, including intestinal hemorrhage, intestinal perforation, and neuropsychiatric abnormalities [4]. Detected early, typhoid fever can be effectively treated with antibiotics; however, if left untreated, it can be life‐threatening [5].

In 2019, Malawi experienced an estimated 6639 cases, 115 deaths, and 8787 disability‐adjusted life‐years lost to typhoid [6]. In Neno District—Malawi, the situation has grown increasingly alarming due to the recurrent outbreaks of typhoid fever, particularly in communities along the Malawi–Mozambique border since 2009 [1]. Neno District comprises many villages which include Mwingitsa, Enock, Yoyola, Lisungwi, Zalewa, Naminga, Symon to mention a few. According to 2023 Health Management and Information System (HMIS) data from Neno District Health Office (DHO), lower Neno reported common cases of typhoid with a history of the lack of access to tap water, forcing people to rely on water from Shire and Lisungwi rivers [7].

Neno District and bordering areas experienced an outbreak of typhoid fever from May 2009 to September 2010, infecting 748 people and causing 44 deaths, and subsequent years saw recurring cases, some involving multidrug‐resistant strains [3]. This resistance complicates treatment efforts and highlights the urgency of comprehensive community‐focused interventions. Despite multidisciplinary efforts to control typhoid, including distribution of Water Guard for in‐home water treatment and education on safe drinking water, handwashing, personal hygiene, and food safety, there is ongoing recurrence of typhoid outbreaks in most areas of Neno District [3, 8]. Neno DHO‐HMIS data show that typhoid outbreaks have been a recurring issue in Neno District. In 2018, there were 7 reported cases, which rose to 19 cases in 2019, resulting in 13 deaths. The number continued to increase in 2020, with 112 cases and 7 deaths. In 2022 and 2023, the situation had worsened, with 606 cases reported, including 17 deaths. Of these 606 cases, 491 originated from the lower Neno community, whereas 115 were from the upper Neno community [7].

The need for community empowerment strategies cannot therefore be overstated when addressing the challenges posed by typhoid fever. It is not enough to merely treat cases as they arise; instead, proactive measures that empower communities to protect themselves and reduce transmission are essential. It is clear from the range of literature that empowerment strategies are promising in their ability to produce both empowerment and health impacts and empowering initiatives can lead to health outcomes and that empowerment is a viable public health strategy and supports active participation [9, 10, 11]. Engaging the affected communities and giving them the tools and knowledge to combat the disease at its source would potentially result in a substantial reduction in the incidence of typhoid fever and improved overall public health outcomes.

This study aimed to assess the factors driving typhoid transmission in Neno District and improve interventions to mitigate its impact. By focusing on community‐driven solutions, the study seeks to improve public health outcomes and create sustainable strategies to fight typhoid in vulnerable areas like Zalewa, Mwingitsa, Naminga, Enock, and Yoyola.

## 2 Methodology

### 2.1 Study Approach, Design, and Setting

The study used a mixed‐method approach, employing a concurrent design. The study was conducted in Neno District and included five villages, which were Zalewa, Mwingitsa, Naminga, Enock, and Yoyola, located in lower Neno, and two health facilities, including Neno District Hospital and Lisungwi Community Hospital. Neno District Hospital is the main public referral hospital in Neno. These villages were selected on the basis of their high prevalence of typhoid fever between 2022 and 2023. Neno is located in the Southern region of Malawi. The district is predominantly rural, with limited urban development and clean water.

### 2.2 Population, Sampling Frame, and Sampling Procedure

The participants for the study included all persons aged 12–44 years and above who reported to Neno District Hospital and Lisungwi Community Hospital for treatment of *S*. Typhi between 2022 and 2023, as well as those who had never been affected by the disease in the five villages. Cases were defined as individuals residing in Neno District, specifically from the five villages with the highest number of reported cases between 2022 and 2023, who presented for medical care at Neno District Hospital or Lisungwi Community Hospital. These individuals were clinically diagnosed with *S*. Typhi infection and had laboratory confirmation through culture and/or positive results from Rapid Diagnostic Tests (RDTs), with the majority being diagnosed using RDTs. Controls were defined as individuals residing in Neno District, specifically from the same five villages with the highest number of reported cases between 2022 and 2023, who did not have symptoms suggestive of *S*. Typhi infection and/or tested negative for *S*. Typhi through culture and/or RDTs during the same period.

### 2.3 Data Collection and Sources

Data collection involved a combination of surveys and interviews for quantitative and qualitative data, respectively. The data were collected from two health facilities in Neno to guide case follow‐up and from various households using a well‐structured and unstructured questionnaire. The structured questionnaire provided standardized responses for statistical analysis, whereas unstructured questionnaire allowed participants to share more detailed opinions and experiences in their own words. The questionnaire was designed to gather detailed information from both cases and controls about the demographic data and factors contributing to the transmission of typhoid fever.

For persons aged below 18, we interviewed the parents or guardians. In households with multiple detected cases, the first case was enrolled in the study. Data from the cases were collected through follow‐up visits after their recovery. Using hospital records, we first identified the cases and their households in the villages. Oral informed consent was obtained from all participants during the household visits. Data collectors, accompanied by healthcare workers, then conducted face‐to‐face interviews at the participants’ homes. A combination of structured and unstructured questions was used to collect both quantitative and qualitative information. Controls were selected from the same villages where the cases were located. Only one individual per household was enrolled as a control.

### 2.4 Data Collection Instrument

Open‐ and close‐ended questions were used. The tools were developed by the researcher, reviewed by experts in the field, and then pretested in a different district before employing it. The questionnaires were designed to protect respondents’ privacy by omitting their names, helping to alleviate any fears and build their confidence. To ensure ease of understanding, questions were presented in the local language (Chichewa), and the responses were later translated into English for analysis. The researcher maintained privacy, and strictly personal interviews were conducted while respondents answered the questions. At the same time, the researcher assured the respondents that the information given would be kept confidential. Adequate time was given to attain information from each respondent.

### 2.5 Sample Size

According to data from the entire Neno District Hospital, a total of 606 typhoid cases, including 17 deaths, were recorded between 2022 and 2023 across all villages. This study focused only on five villages with the highest prevalence of typhoid cases. These five villages had a combined population of 356 cases of typhoid fever. To determine the appropriate sample size for the study. Taro Yamane's formula was used to ensure accurate representation of the population within the given constraints. Taro Yamane formula used was *n* = $\frac{N}{1+N{\left(e\right)}^{2}}$ where *n* is the sample size, *N* is the total population, and *e* is the margin of error or precision, which mostly are 0.01, 0.05, and 0.10, and in this study 0.05 was used [12]:

$$n=\frac{N}{1+N{\left(e\right)}^{2}}=n=\frac{356}{1+356{\left(0.05\right)}^{2}}=188$$

Although Taro Yamane's formula calculated a sample size of 188, a total of 200 cases were interviewed to ensure broader representation and account for potential non‐responses or incomplete data. In addition to the 200 cases, the study also interviewed 200 controls.

### 2.6 Sampling Technique

Purposive sampling technique was used to select cases in the affected villages. These cases were initially identified through hospital records and then followed up in their respective villages. Controls were equally selected purposively from the same villages where cases were identified.

### 2.7 Data Analysis and Data Presentation

Quantitative data were analyzed using Statistics Training Analysis Translation Assistance (STATA) version 17 (Stata Corp, College Station, Texas, USA). Data were presented in the form of tables, figures, and percentages. Descriptive analyses were performed to describe characteristics of study participants and were presented as frequencies and percentages for categorical data. Associations between variables were assessed using odds ratios (ORs) to measure the strength of associations. Logistic regression analysis was employed to calculate adjusted ORs. ORs were used to compare the likelihood of exposure to specific risk factors between both cases and controls (combined) with a reference group serving as the baseline for comparison. The reference group was defined as without or with less risk factor or lowest exposure category. To determine the level of confidence in the results, a *p* value of <0.05 was considered statistically significant. Qualitative data were transcribed and formed the basis for the qualitative analysis. The findings of qualitative data analysis were typically reported in a narrative form, accompanied by illustrative quotes from participants to support the themes and patterns identified.

### 2.8 Inclusion and Exclusion Criteria

The inclusion criteria focused on individuals aged 12 years and above, comprising typhoid fever cases identified between 2022 and 2023 in the five selected villages with the highest number of cases, as well as controls from the same villages who were not infected during the same period. For households where more than one case was detected during this period, only the first identified case was enrolled. If the first case was unavailable, one of the other detected cases present during the assessment in that household was enrolled. For each case, one to three neighborhood controls were selected from different households. These controls were chosen either from households located approximately 100 m away or from more distant households about 150 m from the case household. We excluded individuals who, despite having a history of positive or negative results, did not reside in the selected villages between 2022 and 2023 and had only moved to the villages by the time of the assessment, as they originally lived in other areas.

### 2.9 Ethical Consideration

Ethical approval was obtained from the University of Livingstonia Research and Ethics Committee (UNILIAREC) with approval number UNILIA‐REC/UGS/06/2024. Clearance to conduct the study was obtained from authorities of Neno District Hospital and Lisungwi Community Hospital. Clearance was also obtained from the village heads of Zalewa, Mwingitsa, Naminga, Enock, and Yoyola. Participants consent was obtained through signed consent forms, confirming their willingness to participate in the study. For persons aged below 18, consent was obtained from parents or guardians who were interviewed as part of the study.

## 3 Results of the Study

### 3.1 Demographic Data for Cases and Controls

Both cases and controls showed a similar gender distribution, with females constituting 59% and 41% male of the participants in each group. A notable difference in age distribution was observed, with individuals aged 35–44 making up a higher proportion of cases (35.5%) compared to controls (25.5%), whereas age groups between 12 and 20 were represented with 18.5% for cases and 21.5% for controls. The majority of respondents, both cases and controls, were from Zalewa, accounting for 50% of the participants, followed by Mwingitsa with 16%, Enock 12%, Naminga 11.5%, and Yoyola 10.5%. Educational levels among participants also showed interesting patterns. Primary education was the predominant level among both cases (69%) and controls (68.5%). However, a notable disparity existed in tertiary education levels, with 5% of cases versus 1% of controls having tertiary education. At the secondary level, both cases and controls were represented equally with 25% each. *Refer to* Table 1.

**TABLE 1:** Demographic information for cases and controls, Neno District Hospital and Lisungwi Community Hospital—2022–2023.

Variable	Frequency (*n*) for cases	Percent for cases	Frequency (*n*) for controls	Percent for controls
**Gender**				
Female	118	59	118	59
Male	82	41	82	41
**Age**				
12–20	37	18.5	43	21.5
21–34	54	27.	77	38.5
35–44	71	35.5	51	25.5
>44	38	19	29	14.5
**Village**				
Zalewa	100	50	100	50
Mwingitsa	32	16	32	16
Naminga	23	11.5	23	11.5
Enock	24	12	24	12
Yoyola	21	10.5	21	10.5
**Education**				
Primary	138	69	137	68.5
Secondary	51	25.5	51	25.5
Tertiary	10	5	2	1
None	1	0.5	10	5
**Employment**				
Employed	14	7	11	5.5
Self‐employed	124	62	128	64
Unemployed	62	31	61	30.5
**Source of income**				
Farming	134	67	135	67.5
Salaries	13	6.5	13	6.5
Business	29	14.5	30	15
None	24	12	22	11
**Marital status**				
Married	161	80.5	161	80.5
Single	25	12.5	25	12.5
Divorced	4	2	4	2
Widow	10	5	10	5

### 3.2 Factors Associated With Transmission of Typhoid Fever in Neno District

#### 3.2.1 Demographic Factors: Age, Education, Employment, and Marital Status

Participants aged 21–34 years were significantly more likely to contract typhoid (OR: 2.87; 95% confidence interval [CI]: 1.0–7.9, *p* = 0.042), whereas those participants aged 12–20 were less significant (OR: 1.15; 95% CI: 0.4–3.1, *p* = 0.774) compared to those above 44 years. Participants in the age group of 35–44 were also more likely associated with typhoid fever (OR: 2.79; 95% CI: 0.9–8.6, *p* = 0.073). Regarding education levels, participants who did not attend any level of education had lower odds (OR: 0.10; 95% CI: 0.01–1.2, *p* = 0.070) compared to primary level. Similarly, those who attended secondary education had lower odds (OR: 0.86; 95% CI: 0.4–1.6, *p* = 0.643), whereas tertiary education level had higher association of contracting typhoid (OR: 3.52; 95% CI: 0.6–19.4, *p* = 0.148) compared to the reference group (primary). *Refer to* Table 2.

**TABLE 2:** Summary of different factors associated with transmission of typhoid fever—Neno District Hospital and Lisungwi Community Hospital—Malawi—2022–2023.

Variable	Controls *n* = 200 (%)	Cases *n* = 200 (%)	Odds ratio	95% CI	*p* value
**Age**					
>44	29 (14.5)	38 (19)	Ref	Ref	Ref
35–44	51 (25.5)	71 (35.5)	2.79	0.9–8.6	0.073
21–34	77 (38.5)	54 (27)	2.87	1.0–7.9	0.042
12–20	43 (21.5)	37 (18.5)	1.15	0.4–3.1	0.774
**Education**					
Primary	137 (68.5)	138 (69)	Ref	Ref	Ref
Secondary	51 (25.5)	51 (25.5)	0.86	0.4–1.6	0.643
Tertiary	2 (1)	10 (5.00)	3.52	0.6–19.4	0.148
None	10 (5)	1 (0.5)	0.10	0.01–1.2	0.070
**Employment**					
Employed	11 (5.5)	14 (7)	Ref	Ref	Ref
Self‐employed	128 (64)	124 (62)	1.59	0.4–7.2	0.545
Unemployed	61 (30.5)	62 (31)	2.21	0.4–11.1	0.336
**Marital status**					
Married	161 (80.5)	161(80.5)	Ref	Ref	Ref
Single	25 (12.5)	25 (12.5)	2.01	0.5–7.4	0.295
Divorced	4 (2)	4 (2)	0.47	0.1–2.9	0.416
Widow	10 (5)	10 (5)	1.17	0.3–4.3	0.806
**Handwashing after toilet**					
Wash hands with soap	89 (44.5)	109 (54.5)	Ref	Ref	Ref
Wash hands but without soap	50 (25)	56 (28)	0.48	0.2–0.09	0.02
Don't wash hands	61 (30.5)	35 (17.5)	0.39	0.2–0.8	0.006
**Where do you dispose your waste**					
In the rubbish pit	138 (69)	112 (56)	Ref	Ref	Ref
Along the road	17 (8.5)	25 (12.5)	0.61	0.2–1.5	0.293
Anywhere	45 (22.5)	63 (31.5)	0.61	0.3–1.2	0.138
**Where do you get your meals**					
Prepare and eat at home	167 (83.5)	140 (70)	Ref	Ref	Ref
Eat from the restaurant	19 (9.5)	33 (16.5)	3.43	1.1–10.6	0.032
Ready‐to‐eat meals sold along the roads	14 (7.00)	27 (13.50)	3.04	1.3–7.18	0.011
**Water sources**					
Borehole	78 (39)	49 (24.5)	Ref	Ref	Ref
River	122 (61)	151 (75.5)	0.56	0.3–0.97	0.040
**Where do you keep water**					
Cray pots	39 (19.5)	15 (7.5)	Ref	Ref	Ref
Plastic buckets	86 (43)	163 (81.5)	7.98	3.6–17.6	0.001
Jerry cans	75 (37.5)	22 (11)	0.67	0.3–1.7	0.393
**Distance from water source to nearest latrine**					
Above 30 m	77 (38.5)	137 (68.5)	Ref	Ref	Ref
Less than 30 m	123 (61.5)	63 (31.5)	7.01	3.8–12.8	0.001

Abbreviation: CI, confidence interval.

#### 3.2.2 Hygiene and Waste Disposal Factors

Individuals who washed their hands without soap were associated with lower odds of contracting typhoid fever (OR: 0.48; 95% CI: 0.2–0.9, *p* = 0.02) compared to those who washed their hands with soap. Similarly, individuals who did not wash their hands also had lower odds (OR: 0.39; 95% CI: 0.2–0.8, *p* = 0.006). Disposing of waste along the road and anywhere also had lower odds of contracting typhoid fever (OR: 0.61; 95% CI: 0.2–1.5, *p* = 0.293 and OR: 0.61; 95% CI: 0.3–1.2, *p* = 0.138), respectively, compared to the reference group (use of rubbish pit). *Refer to* Table 2.

#### 3.2.3 Food Hygiene and Consumption Practices

Regarding participants who ate meals from restaurants and ready‐to‐eat meals sold along the roads. Specifically, eating from restaurants was presented with a higher association (OR: 3.43; 95% CI: 1.1–10.6, *p* = 0.032). Similarly, ready‐to‐eat meals sold along the roads (OR: 3.04; 95% CI: 1.3–7.18, *p* = 0.011) compared to those that prepared and ate at home. *Refer to* Table 2.

#### 3.2.4 Water and Sanitation Factors: Water Source, Water Storage, and Distance From Water Source to Latrine

Those who used river water were represented with lower odds of contracting the typhoid fever (OR: 0.56; 95% CI: 0.3–0.97, *p* = 0.040) compared to those who used borehole water. Participants who stored their water in plastic buckets were represented with higher odds of contracting typhoid fever (OR: 7.98; CI 3.6–17.6, *p* = 0.001) compared to those who used cray pots. In contrast, storing water in jerry cans was not associated with higher odds of transmission of typhoid fever (OR: 0.67; 95% CI: 0.3–1.7, *p* = 0.393) compared to those who used clay pots. Regarding individuals who collected water from a river or borehole located less than 30 m from a latrine, they were 7.0 times more likely to contract typhoid (OR: 7.01; 95% CI: 3.8–12.8, *p* = 0.001) than those whose water source was about 30 m away. *Refer to* Table 2.

### 3.3 Level of Knowledge on Typhoid Fever

As shown in Table 3, nearly a third of participants (31.5%; *n* = 126), comprising both cases and controls, identified contaminated food and water as the primary sources of transmission. Unsafe food handling and close contact with an infected person were noted by 21.3% (*n* = 85). Poor hygiene practices and close contact with a person who has typhoid were recognized by 16.25% (*n* = 65), the same percentage that acknowledged poor sanitation and contaminated water as factors. However, a notable number (14.6%; *n* = 59) admitted to not knowing how typhoid fever spreads.

**TABLE 3:** Respondents’ knowledge on the spread of typhoid fever, Neno, 2023.

Variable	Frequency (*n*)	Percentage
I don't know	59	14.6
Contaminated food and water	126	31.5
Poor hygiene practices and close contact with a person who has typhoid	65	16.3
Poor sanitation and contaminated water	65	16.3
Unsafe food handling and close contact with a person who has typhoid	85	21.3

### 3.4 Awareness of Typhoid Fever Preventive Measures

The majority, 85.5% of respondents, were aware of the preventive measures for typhoid fever. In contrast, only 14.5% indicated that they were not aware of these measures. Of the 85.5%, who were aware of preventive measures, 82% (*n* = 163) were cases, whereas 89% (*n* = 178) were controls.

### 3.5 Community Empowerment Strategies

#### 3.5.1 Impact of Education Intervention in Community on Typhoid Fever Prevention Practices

Out of 400 participants, 27.5% (*n* = 110) said that education can help them in promoting proper handwashing techniques to prevent spread of the bacteria. One respondent noted: “*Education on prevention can help us learn proper handwashing techniques and the right way to wash hands to minimize the spread of the disease*.” However, 21% (*n* = 84) said that education can help them to increase awareness on safe food handling. Respondent EF commented: “*Unsafe food handling increases the chances of typhoid. Education on proper food preparation and storage is crucial.”* Additionally, 26% (*n* = 104) said education can help in promoting the installation and management of toilets and rubbish pits. Respondent DX stated: “*Routine education campaigns can help us prevent typhoid by working with village heads to enforce rules, such as having a toilet and rubbish pit.”* Although 25.5% (*n* = 102) said that education can help in increasing awareness on importance of using boiled water to prevent infection, one respondent stated, “*Education can help us understand the importance of boiling water, especially when some dislike chlorine‐treated water.” Refer to* Table 4.

**TABLE 4:** Community empowerment strategies on prevention of spread of transmission of typhoid fever in Neno District—2023.

Variables	Frequency (*n*)	Percent
**Impact of education intervention in community on typhoid fever prevention practices**		
Promote proper handwashing techniques to prevent the spread of the bacteria	110	27.5
Promote the installation and management of toilets and rubbish pits	104	26
Increases awareness on importance of use of boiled water to prevent infection	102	25.5
Increases awareness on safe food handling	84	21
**Community involvement as an empowerment strategy in controlling transmission of typhoid fever**		
Help in behavior change	103	25.8
Helps in early healthcare‐seeking behavior	87	21.8
Encourage use of preventive measures	105	26.3
Provide local knowledge and awareness	105	26.3
**Actions to be taken by community**		
Ensure that food is cooked thoroughly and eat while it is hot	62	15.5
Everyone should have toilet and rubbish pit	111	27.8
Practicing proper hygiene and sanitation	112	28
Treat water before drinking	115	28.8

#### 3.5.2 Community Involvement as an Empowerment Strategy in Controlling Transmission of Typhoid Fever

Of the 400 participants, 26.3% (*n* = 105) said that if community can be involved and take part in control of transmission of typhoid fever this can help to encourage them to use preventive measures and provide local knowledge and awareness, respectively. Respondent FG stated, “*small groups can enable us to share health information, like using 5 liter bottles as handwashing equipment in toilets.”* However, 21.8% (*n* = 87) said that it helps in early healthcare‐seeking behavior. Furthermore, 25.6% (*n* = 103) of participants said that community involvement can help in behavior change. *Refer to* Table 4.

#### 3.5.3 Actions to be Taken by the Community to Prevent Spread of Typhoid Fever

Out of 400 participants, 28.6% (*n* = 115) stated that water should be treated before drinking. Respondent BL shared, “As we don't have access to clean water sources like taps, we use river water, which should be treated before use. Treating water with chlorine or boiling will help prevent the spread of typhoid fever and other diseases.” Although 15.5% (*n* = 62) of participants said that they should ensure that food is cooked thoroughly and eat while it is hot, respondent NN explained, “Undercooked vegetables, especially cabbage, can increase the spread of typhoid fever. I ensure my family's food is well‐cooked and served hot to help prevent the disease.” However, 28% (*n* = 112) of participants said that they should be practicing proper hygiene and sanitation. As one respondent explained, “Children should be well cared for, ensuring they wash their hands before eating and clean fruits properly. This can help prevent the spread of the disease in our community.” Similarly, 27.6% (*n* = 111) of participants said that everyone should have toilet and rubbish pit. One participant reflected, “I used to think toilets weren't important, but after health education, I realized their value. Since we started using a toilet and rubbish pit, none of my household members have suffered from typhoid fever.” Refer to Table 4.

## 4 Discussions

### 4.1 Demographic Factors

The study revealed no significant gender differences in typhoid fever transmission among cases from the five selected villages, with 59% being female and 41% male of those sampled. The findings suggest that the transmission of typhoid fever was not influenced by gender in these selected villages and the representation of the participants. However, other studies expressed varied results: Such as a study in Bangladesh observed higher infection rates among males population than females [13]; on the other hand, a study in Ghana found females more affected (66.9%) than males (33.1%) [14]. These findings emphasize the idea that both males and females are both at risk.

Participants aged 21–34 years were significantly 2.87 times more likely to contract the disease over 44 years (OR: 2.87; 95% CI: 1.0–7.9, *p* = 0.042). This study finding suggests that individuals in this age range are active in their communities and workplace, run small businesses (moving from one place to another), and have increased social interaction which might expose them to poor sanitation environments hence, at increased risk of exposure. This aligns with global studies indicating that younger age groups, particularly those in their early 20s to mid‐30s, are at higher risk for typhoid fever [1, 14].

### 4.2 Hygiene and Waste Disposal Factors

Participants who washed hands after using the toilet without soap were 0.48 times of experiencing typhoid fever (OR: 0.48; 95% CI 0.2–0.9, *p* = 0.02) compared to those who used soap. Interestingly, those that did not wash their hands after using the toilet had lower odds of contracting typhoid fever (OR: 0.39; 95% CI 0.2–0.8, *p* = 0.006) compared to those who washed hands with soap. Both of these findings were statistically significant with *p* < 0.05. These unexpected findings may reflect factors such as individuals adopting other protective behaviors such as use of hand sanitizer, avoiding street food, and accessing treated water just to mention a few. Despite these findings, proper handwashing with soap remains a crucial preventive measure as supported by a study in Malawi at Kaziwiziwi coal mine that found not having a facility for handwashing practices after toilet use had a higher association for contracting typhoid fever [15]. Likewise, a study in Fiji found that using soap for handwashing was associated with lower odds for typhoid fever [16]. The findings in the present study suggest they need for comprehensive education on proper handwashing practices and techniques. Thus, further investigation is needed in Neno District to explore handwashing practices after toilet use. This further investigation is alluded to by a previous study that interestingly reported that increased risk was among people with knowledge about handwashing and practicing before cooking and after toilet use [17], hence the need for further investigation.

### 4.3 Food Hygiene and Consumption Practices

Eating meals at restaurants (OR: 3.43; 95% CI: 1.1–10.6, *p* = 0.032) and ready‐to‐eat meals sold along the roads (OR: 3.04; 95% CI: 1.3–7.18, *p* = 0.011) were statistically significant (*p* < 0.05) and associated with higher odds of typhoid fever than preparing and eating meals at home. Those who usually eat at restaurants or consume ready‐to‐eat meals sold along the roads are often business people who travel between villages, spending several days away or returning home late at night. Previous studies have highlighted that most street vendors have knowledge of the required hygiene measures, even though they rarely use this knowledge and break hygiene rules at the site of food preparations and distribution [18, 19]. The present findings support strengthening food safety regulations and monitoring compliance to standards in restaurant and food vendors [20].

### 4.4 Water and Sanitation Factors

Clean and safe drinking water is a fundamental requirement for preventing waterborne diseases. In this study area, access to clean water is a common problem forcing people to rely on rivers and boreholes and usually household rely on stored water. Those who used river water had statistically significant lower odds of contracting typhoid fever (OR: 0.56; 95% CI: 0.3–0.97, *p* = 0.040) compared to those who relied on borehole. This result contradicts previous studies [21, 22] which associate river water with contamination and disease risk. The finding in this study may reflect limited variations in water safety or treatment, such as individual using river water might be treating the water before use like boiling or adding disinfectant, reducing the risk of typhoid fever transmission. Boreholes are usually associated with safe water. On the other hand, findings in this study suggest possible contamination of boreholes due to poor and improper handling during maintenance and lack of disinfectants. These findings highlight the need for further investigation into water treatment and sanitation practices among different users in this study area.

Storing water in plastic buckets statistically significantly increased the risk for typhoid fever (OR: 7.98; 95% CI: 3.6–17.6, *p* = 0.001) compared to clay pots, likely due to contamination risks from wide opening, poor coverage, and poor water storage practices. The finding echoes those of Pal et al. who emphasized the importance of promoting and strengthening safe water practice [23]. Individuals who collected water from a river or borehole located less than 30 m from a latrine were 7.0 times more likely to contract typhoid than those whose water source was more than 30 m away (OR: 7.01; 95% CI: 3.8–12.8, *p* = 0.001). The result was statistically significant with *p* < 0.05. The results highlight the importance of continued community awareness in Neno about risk of collecting water near potential contaminated areas/sources without treatment and having improved sanitation infrastructure, including latrines with recommendation from health experts. This is echoed by a previous study that highlighted consumers should treat groundwater and seek expert's advice before sinking groundwater [24].

### 4.5 Level of Knowledge on Typhoid Fever

The data revealed varying levels of awareness among respondents regarding the spread of typhoid fever. A notable 14.6% of respondents admitted not knowing how typhoid fever spreads, and nearly a third of participants (31.5%; *n* = 126), comprising both cases and controls, identified contaminated food and water as the primary sources of transmission. These results indicate that participants in the present study had a considerable level of understanding of the primary sources of typhoid fever transmission, whereas a significant minority remained unaware of critical factors. This aligns with a previous study that reported despite good knowledge among the majority (63.8%), others lacked understanding that eating contaminated food (7.3%) and drinking contaminated water (12.1%) can transmit typhoid fever [25]. A similar study conducted among female heads in Neno District in 17 villages reported that 51% of participants were unaware that typhoid fever can be caused by drinking unsafe water, whereas 75% lacked knowledge that consuming unsafe food can also lead to the disease [3]. The findings in the present study suggest strengthening mass media campaigns to disseminate easy‐to‐understand messages on typhoid fever transmission and prevention targeting everyone.

Regarding preventive measures, the study found that majority, 85.5% of respondents (both cases and controls), were aware of the preventive measures for typhoid fever. This high awareness level suggests that public health campaigns have considerably effectively disseminated information. However, the minority (14.5%) lacking awareness suggests the need for ongoing education and community proactive actions in Neno District. Similar findings were reported by a previous study that emphasized the importance of continuous community engagement and education to maintain and improve awareness levels and actions [9]. The findings in this study also suggest that interventions should prioritize strengthening knowledge on key preventive measures and enhancing awareness of typhoid fever, including encouraging proactive actions.

### 4.6 Community Empowerment Strategies

Education emerged as a critical factor in typhoid fever prevention. The findings indicate that participants in this study had a considerable knowledge on the impact of education in community on typhoid prevention. These findings align with a previous research that found education programs significantly contribute to promoting handwashing, safe food handling, and using boiled water, which are essential preventive measures [9].

Community involvement was identified as pivotal in controlling the transmission of typhoid fever by considerable participants in the present study. To enhance these efforts, this study suggests that health education campaigns, community‐driven initiatives, and collaborations with local leaders can further strengthen awareness, empower individuals, and sustain long‐term preventive practices to promote behavior change. This is in support with previous study findings that highlighted the impact of community‐based interventions and education programs on reducing typhoid transmission rates [9]. Additionally, the study identified considerable actions that community members could adopt to prevent the spread of typhoid fever, and the findings in this present study highlight the critical role of sanitation infrastructure improvements, water treatment, hygiene, and food preparation practices in controlling typhoid fever.

## 5 Limitations

While eating at restaurants, consuming ready‐to‐eat meals sold along the roads, and using water stored in plastic buckets, as well as the distance of less than 30 m from water sources to latrines, were associated with an increased risk of typhoid fever, our study had some limitations. We did not collect or analyze food and water samples to confirm the presence of *S*. Typhi, which limits our ability to establish direct evidence linking these factors to the infection. Observations of handwashing practices were not performed (only associations were identified, not cause‐and‐effect relationship). Therefore, our findings should be interpreted with caution. The study does not isolate the association between exposure and disease but instead gives a broader perspective of exposure trends. Participants may not have accurately remembered the past exposure. This recall bias could affect the reliability of the data, leading to inaccuracies that may influence the interpretation of the results. Only five villages in Neno with high prevalence rates were selected, which may restrict the generalizability of the findings to other villages in the district. Likewise, there was a bias in the selection of the age group to include only older children and adults (12 years old and above), and this may also limit the generalizability of the findings.

## 6 Conclusions and Recommendations

The findings reveal varying gaps in community knowledge about typhoid fever. Health education, community involvement, and fostering community action were considerably identified as pivotal empowerment strategies in preventing and controlling typhoid fever. Eating food from restaurant and ready‐to‐eat meals sold along the roads, storing water in plastic buckets, young adults aged 21–34 years, and distance of less than 30 m from water sources to latrines appear to increase odds for typhoid fever transmission in this study area. As part of the efforts to achieve Sustainable Development Goal 3, target 3.3 that aims to combat hepatitis, waterborne diseases, and other communicable diseases by 2030, implementation and strengthened measures that are likely to contribute to the prevention and control of typhoid fever in Neno District according to the study findings includes improved sanitation and hygiene practices at all levels; water treatment before use; strengthened food safety regulations and monitoring compliance in restaurant and food vendors; sanitation infrastructure improvement; strengthened routine community awareness and involvement; particularly interventions targeting among young adults aged 21–34 years in the interventions, together with uptake of typhoid conjugate vaccination. These findings highlight the need for further investigation to explore water storage and treatment practices, food safety regulations, and monitoring compliance among different users in this study area.

## Author Contributions

Priscilla Talabuku, Atusaye Mwalwanda, Tawonga Mwase‐Vuma, Elton Chavura, and Balwani Chingatichifwe Mbakaya contributed substantially to the conception and design. Priscilla Talabuku, Atusaye Mwalwanda, Tawonga Mwase‐Vuma, Elton Chavura, and Balwani Chingatichifwe Mbakaya analy**z**ed and interpreted the data. Priscilla Talabuku, Atusaye Mwalwanda, Tawonga Mwase‐Vuma, Elton Chavura, and Balwani Chingatichifwe Mbakaya drafted the manuscript and revised it. Priscilla Talabuku, Atusaye Mwalwanda, Tawonga Mwase‐Vuma, Elton Chavura, and Balwani Chingatichifwe Mbakaya approved the manuscript for submission.

## Ethics Statement

Ethical approval was obtained from the University of Livingstonia Research Ethics Committee (UNILIAREC) with approval number UNILIA‐REC/UGS/06/2024.

## Consent

Participants’ consent was obtained through signed consent forms, confirming their willingness to participate in the study. For persons aged below 18, consent was obtained from parents or guardians who were interviewed as part of the study.

## Conflicts of Interest

The authors declare no conflicts of interest.

## Data Availability

Data are available on request through the corresponding author.

## 1

Questionnaire Consent Form This study seeks to understand the factors contributing to the transmission of typhoid fever in the Lower Neno community of Neno District. You have been selected to participate, and your input will help identify key risk factors among patients attending Neno District Hospital and Lisungwi Community Hospital. The findings will support efforts to improve healthcare services in the area and inform future research. Participation is voluntary, and all responses will be kept confidential. You may choose not to answer any question that makes you uncomfortable and can withdraw at any time without affecting any services you receive. Signature/thumbprint of the respondent……………………………………. . Date………. . /………. . /……………. . SECTION A: DEMOGRAPHIC DATA (Tick the correct option) 1. Age a) 12–20 years b) 21–34 years c) 35–44 years d) More than 44 years 2. Sex a) Male b) Female 3. Marital status a) Single b) Married c) Divorced d) Widowed 4. Education level a) Primary b) Secondary c) Tertiary d) None 5. Employment status a) Employed b) Self‐employed c) Unemployed 6. Main source of income a) Farming b) Salaries c) Wages d) Business e) Others (specify): …………………………………………………………………………………………. SECTION B: SANITARY AND HYGIENIC FACTORS (Tick the correct option) 7. Do you have a latrine at home? a) Yes b) No 8. If No, where do you dispose of fecal matter? a) Nearby bush b) In polythene bags c) Neighbor's pit‐latrine d) Others (specify): ……………………………………………………………………………………………. . 9. How do you control flies from your latrine? a) Cover the hole b) Use VIP latrine c) Spray with insecticides d) Do not control flies e) Others (specify): ………………………………………………………………………………………………. 10. Do you wash hands with soap after using the latrine? a) Yes b) No c) Wash, but without soap 11. Where do you dispose of your rubbish? a) Rubbish pit b) Along the road c) Anywhere SECTION C: FOOD AND PERSONAL HYGIENE FACTORS (Tick the correct option) 12. Where do you get your meals? a) Prepare and eat at home b) Eat from the restaurant c) Buy already cooked food along the road d) Others (specify): ………………………………………………………………………………………………………. 13. How often do you wash your hands before preparing food? a) Always b) Sometimes c) Not at all 14. How do you store cooked food for later use? a) Well‐covered in a pot b) In a refrigerator c) Leave food uncovered d) Others (specify): ………………………………………………………………………………………………………. 15. How do you prepare fresh fruits before eating? a) Wash with clean water b) Peel c) Eat straight away without washing/peeling SECTION D: WATER‐RELATED FACTORS (Tick the correct option) 16. Where do you get your water? a) Tap b) Borehole c) Well d) Spring e) Others (specify): ……………………………………………………………………………………………………… 17. What do you do to make water safe for drinking? a) Boil b) Drink without boiling c) Treat with tablets (chlorine, Water Guard, etc.) d) Others (specify): ……………………………………………………………………………………………………… 18. Where do you keep your water? a) Jerry cans b) Clay pots c) Plastic buckets d) Others (specify): ……………………………………………………………………………………………………. 19. How often do you clean your water containers? …………………………………………………………………………. 20. What is the distance of the nearest latrine from your water source? a) Less than 30 m b) About 30 m c) More than 30 m SECTION E: LEVEL OF KNOWLEDGE ON TYPHOID FEVER 21. What is your understanding of typhoid fever, its causes, and symptoms? ………………………………………………………. 22. Have you or anyone you know ever been diagnosed with typhoid fever? a) Yes b) No 23. Are you aware of any preventive measures to reduce the risk of typhoid fever transmission? a) Yes b) No 24. Where do you usually seek information about health issues such as typhoid fever? a) Hospital b) Church c) Friends d) Others (specify): …………………………………………………………………………………………………………. 25. Have you received any education/guidance on typhoid fever from health authorities or organizations? a) Yes b) No 26. Do you think there is a need for more information/awareness programs on typhoid fever in your community? a) Yes b) No SECTION F: COMMUNITY EMPOWERMENT STRATEGIES 27. Are there any existing community health programs related to typhoid fever prevention in Lower Neno? a) Yes b) No 28. What role does education play in preventing the spread of typhoid fever? How can it be improved in your community? ……………………………………………………………………………………………. 29. How effective do you think community involvement is in controlling typhoid fever transmission? ……………………………………………………………………………………………………………………. 30. What challenges do you face in accessing healthcare facilities or information about typhoid fever in Lower Neno? ………………………………………………………………………………………………………… 31. What specific strategies can be taken by the community to effectively prevent typhoid fever spread? ……………………………………………………………………………………………………………………………. .
